# Impaired Gastrointestinal Motility and Worsening Heart Failure in Patients Receiving Trans-Catheter Aortic Valve Replacement

**DOI:** 10.3390/jcm13154301

**Published:** 2024-07-23

**Authors:** Teruhiko Imamura, Nikhil Narang, Ryuichi Ushijima, Mitsuo Sobajima, Nobuyuki Fukuda, Hiroshi Ueno, Koichiro Kinugawa

**Affiliations:** 1 The Second Department of Internal Medicine, University of Toyama, Toyama 930-0194, Japan; 2 Advocate Christ Medical Center, Oak Lawn, IL 60453, USA

**Keywords:** laxative, heart failure, hemodynamics, aortic stenosis

## Abstract

**Background:** Impaired gastric motility in the form of constipation may often occur in elderly patients with chronic heart failure. Candidates for trans-catheter aortic replacement (TAVR) are of old age and have multiple comorbidities, probably including constipation. However, the clinical implication of a history of constipation in patients receiving TAVR remains unknown. **Methods:** Patients who underwent TAVR at our large academic center between 2015 and 2022 were eligible. The prognostic impact of the prescribed laxative type and number, which was assumed as the severity of constipation, on the incidence of death or heart failure readmission two years after index discharge was investigated. **Results:** A total of 344 patients were included. Median age was 85 years, and 99 patients were men. Patients with any laxatives (*N* = 166) had higher systolic blood pressure, higher plasma B-type natriuretic peptide levels, and a lower prescription rate of renin–angiotensin system inhibitors at the time of index discharge after TAVR (*p* < 0.05 for all). The number of laxative types was independently associated with the composite primary outcome with an adjusted hazard ratio of 1.83 (95% confidence interval 1.27–2.63, *p* = 0.001) with a cutoff of one type of laxative used, which significantly stratified the 2-year cumulative incidence of the primary outcome (18% versus 7%, *p* = 0.001). **Conclusions:** The presence of constipation was associated with worse clinical outcomes following TAVR. The prognostic impact of an aggressive intervention for constipation remains a future concern in this cohort.

## 1. Background

Trans-catheter aortic valve replacement (TAVR) is a recognized less invasive trans-catheter intervention for approaching symptomatic severe aortic stenosis, primarily targeting individuals with high operative risk for surgical valve replacement [1]. Advances in clinical outcomes following TAVR have steadily grown due to optimal choices in patient selection, implementation of comprehensive pre-procedural imaging assessments, sophistication of next-generation device designs, and improvement of peri-procedural clinical management [2]. Consequently, TAVR indication has expanded to encompass patients with intermediate and low surgical risk, demonstrating clinical outcomes comparable to surgical valve replacement [3,4]. As a result, the number of TAVR procedures is increasing so far. However, many TAVR candidates are of advanced age, with multiple comorbidities, which may not necessarily be addressed by TAVR alone and may persist even after a successful TAVR procedure. Thus, post-TAVR clinical outcomes may rather be affected by non-cardiovascular clinical problems which need to be considered when determining if the clinical benefits of the procedure will outweigh any imminent progression of other medical comorbidities [5].

Impaired gastric motility in the form of constipation is a common clinical problem in geriatric populations, and it can be exacerbated by general anesthesia, narcotics, immobility, and prolonged hospital stays [6]. It is not surprising that comorbid constipation is prevalent and may be one of the pivotal comorbidities among TAVR candidates, considering the features of this cohort. Heart failure and its related comorbidities, such as chronic kidney disease, together with its management, such as diuretic use, can worsen constipation. Thus, heart failure is directly and indirectly associated with worsening constipation. Conversely, single-center data by Namiuchi and colleagues have demonstrated that the presence of constipation needing laxatives even after propensity matching was associated with a greater than two-fold increase in the risk of heart failure readmission [7]. It can also be postulated that the effects of straining in patients with constipation significantly increase afterload and can potentially contribute to heart failure decompensation [8]. Thus, the presence of constipation can be a major cause of worsening heart failure.

Many TAVR candidates frequently also have heart failure with preserved ejection fraction, partly due to chronic age-related essential hypertension and ventricular hypertrophy, which may be present in the setting of severe aortic stenosis [8]. It is reasonable to postulate that impaired gastric motility, in the form of constipation, which is frequently encountered in geriatric populations, is also present in many patients with age-related severe aortic stenosis. Detailed knowledge of the relationship between constipation and clinical outcomes in this cohort should further improve therapeutic strategies using TAVR; however, few studies have investigated them. In this retrospective study, we investigated the impact of constipation, which was objectively defined as the prescription of laxatives, on the risk of death and heart failure hospitalization among patients who underwent TAVR.

## 2. Methods

### 2.1. Patient Selection

Patients with symptomatic severe aortic stenosis who underwent TAVR at our large academic center between 2015 and 2022 were eligible. Patients who died during the index hospitalization or those who were lost to follow-up were excluded. Patients with critical data missing were also excluded. Written informed consent to being included in the prospective database was procured from all participants. The study protocol received approval from the institutional review board.

### 2.2. Study Design

We conducted this retrospective study using a prospectively collected institutional dataset. Efforts were made to minimize missing data in accordance with institutional protocols for constructing prospective datasets. The consistency and accuracy of the dataset, together with adherence to follow-up, were regularly ensured by independent researchers.

Day 0 was defined as index discharge. The independent variable of the present study was defined as the number of laxative types prescribed at index discharge, instead of the number of drug tablets. We focused on the number of laxative types and not the number of laxative tablets.

Patients were followed up after the index discharge for up to two years. The dependent variable (primary outcome) was defined as death or readmission due to worsening heart failure during the observation period. The indication of heart failure readmission was at the discretion of the attending board-certified cardiologists. Briefly, patients with worsening heart failure who required IV diuretics and/or inotrope therapies during careful in-hospital observation were hospitalized. We aimed to investigate the association between constipation via the surrogate of laxative requirement and the primary outcome.

### 2.3. TAVA Procedure

Patients with severe aortic stenosis, characterized by a peak velocity surpassing 4.0 m/s, a mean pressure gradient exceeding 40 mmHg, or an aortic valve area less than 1.0 cm^2^, were identified as eligible for TAVR procedures. The decision to proceed with TAVR was reached through clinical consensus by the institutional multidisciplinary heart valve team, which included interventional cardiologists, general cardiologists, cardiac surgeons, anesthesiologists, nurses, and imaging specialists. This decision-making process involved comprehensive informed consent discussions and shared decision-making with both patients and their respective relatives.

Patients underwent a standardized TAVR procedure using either the Edwards Sapien XT/Sapien 3 Transcatheter Heart Valve (Edwards Lifesciences, Irvine, CA, USA) or the Medtronic CoreValve/Evolut R Revolving System (Medtronic, Minneapolis, MN, USA). The TAVR procedure was performed under local or systemic anesthesia as determined by the established heart valve team.

The post-TAVR antithrombotic therapy was selected at the discretion of the treating physician. Following a week of in-hospital monitoring and confirmation of the absence of active procedure-related complications following the procedures, patients were finally discharged. After the index discharge, patients underwent routine follow-up appointments at either our outpatient clinic or affiliated institutions, conducted by board-certified cardiologists. Standard laboratory and echocardiographic assessments were performed at least annually, and the findings were also assessed by board-certified cardiologists.

### 2.4. Clinical Data Collection

Baseline characteristics were retrieved from the prospectively constructed institutional comprehensive dataset, consisting of demographics, comorbidities, laboratory results, echocardiographic data, and medication information at the time of index discharge after TAVR. Of note, the number of laxative types was recorded as an independent variable.

Day 0 was defined as the time of index discharge. The primary outcome, comprising death and heart failure readmission, was evaluated during the two-year observation period following the index discharge.

### 2.5. Statistical Assessment

Continuous variables were displayed as medians and interquartile ranges (25th and 75th percentiles), irrespective of the normality of their distribution, while categorical variables were articulated as counts and corresponding percentages. Statistical significance was defined as a two-tailed *p*-value less than 0.05. The statistical analyses were conducted using SPSS Statistics 23 in a standard manner (SPSS Inc., Armonk, IL, USA).

Continuous variables were assumed as non-parametric parameters and compared between the two groups by the Mann–Whitney U test. Categorical variables were compared between the two groups by Fischer’s exact test.

The independent variable was defined as the number of laxative types prescribed at index discharge. The dependent variable (primary outcome) was defined as a composite of death and heart failure readmission. Cox proportional hazard ratio regression analysis was performed to evaluate the impact of laxative type number on the primary outcome. Variables that were considered to be potentially associated with the primary outcome, in addition to the independent variable, were pre-specified and included in the univariable analyses, including age, systolic blood pressure, renal function, B-type natriuretic peptide level, hemoglobin, cardiac systolic function, the presence of atrial fibrillation, and the prescription of anti-heart failure agents. Variables with *p* < 0.05 in the univariable analyses were included in the multivariable analysis.

A receiver operating characteristic analysis was performed to calculate a cutoff of laxative type number for predicting the primary outcome. The cohorts were divided into two groups using the cutoff. The cumulative incidence of the primary outcome during the two-year observation period was compared between the two groups by the log-rank test.

## 3. Results

### 3.1. Baseline Characteristics

A total of 344 patients who underwent TAVR and were discharged alive were included in this retrospective study. Median age was 85 (83, 89) years, and 99 (29%) patients were men. At index discharge, the estimated glomerular filtration rate was 48.6 (34.0, 62.1) mL/min/1.73 m^2^ and plasma B-type natriuretic peptide was 110 (57, 227) pg/mL (Table 1). Aortic stenosis was successfully treated with maximum velocity at aortic valve of 2.1 (1.7, 2.4) m/s and mean pressure gradient at aortic valve of 10 (7, 12) mmHg. Renin–angiotensin system inhibitors were prescribed in 224 (65%) patients, and 154 patients (45%) had loop diuretics.

### 3.2. Laxative Prescription

**Figure 1:**
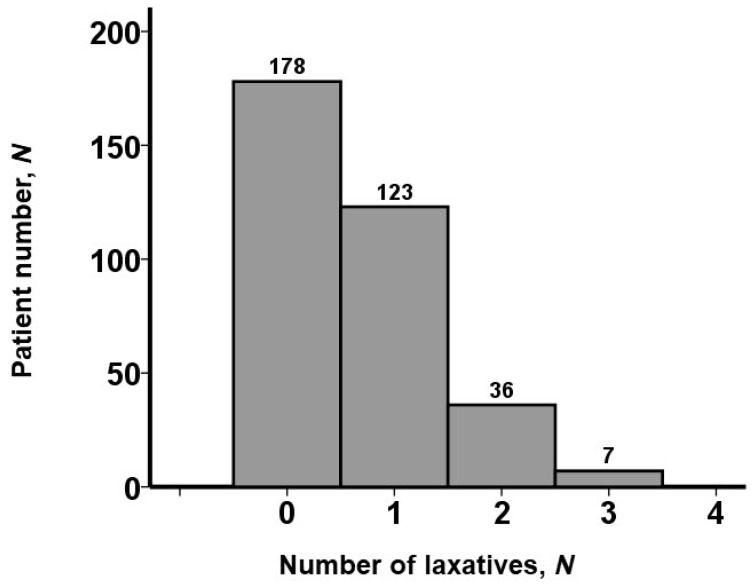
Distribution of laxative type numbers. Approximately half of the patients received no laxatives. No patients received over 3 types of laxatives.

Distributions of the number of laxatives prescribed at index discharge are displayed in Figure 1. Approximately half of the patients (178 [52%]) received no laxative. One type of laxative was prescribed in 123 patients, two types of laxatives were prescribed in 36 patients, and 7 patients received three types of laxatives. No patients received more than three types of laxatives.

Baseline characteristics were compared between those with and without laxatives (Table 1). Patients with laxatives had significantly higher systolic blood pressure and plasma B-type natriuretic peptide levels compared with those not receiving laxatives (*p* < 0.05 for both). Other baseline characteristics did not significantly differ between the two groups.

### 3.3. Prognostic Impact of Laxative Prescription

During the period of 730 (359, 730) days after the index discharge, 35 patients experienced the primary outcome; 29 patients had a heart failure readmission; and 21 patients died within the follow-up observational period (i.e., 29 patients encountered heart failure readmissions and subsequent death, whereas 6 patients died without heart failure readmissions). The 2-year mortality was 6%, the 2-year heart failure readmission rate was 7%, and the 2-year cumulative incidence of the primary outcome was 12%.

The number of laxatives was independently associated with the primary outcome, together with plasma B-type natriuretic peptide levels and the presence of atrial fibrillation, with an adjusted hazard ratio of 1.83 (95% confidence interval 1.27–2.63, *p* = 0.001; Table 2).

The hazard ratio of one type of laxative (*N* = 123) versus no laxative on the primary outcome was 1.90 (95% confidence interval 0.85–4.24, *p* = 0.12). That of two types of laxatives (*N* = 36) versus no laxatives on the primary outcome was 4.84 (95% confidence interval 2.06–11.4, *p* <0.001). That of three types of laxatives (*N* = 7) versus no laxatives on the primary outcome was 2.20 (95% confidence interval 0.28–17.1, *p* = 0.45).

As a breakdown of the primary outcome, the hazard ratio of laxative type number on heart failure readmission was 1.97 (95% confidence interval 1.22–3.21, *p* = 0.006) and 1.62 (1.01–2.60, *p* = 0.047) for all-cause death, respectively.

**Figure 2:**
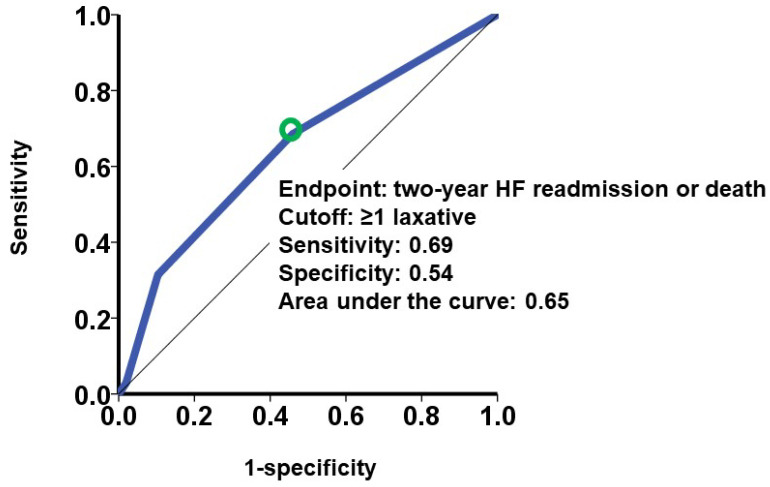
Association between laxative type number and the primary outcome. The primary outcome was defined as heart failure readmission or death during a two-year observation period. The cutoff of laxative type number was calculated by receiver operating characteristic analysis for the primary outcome. A green circle represents the cutoff of laxative type number. The cutoff was calculated as one type of laxative. HF, heart failure.

**Figure 3:**
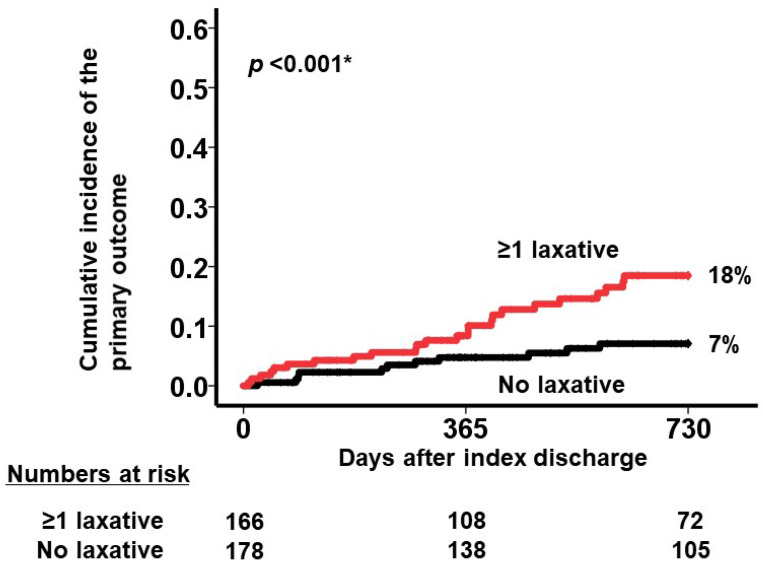
Cumulative incidence of the primary outcome stratified by the prescription of laxative (any laxative prescription [red curve] versus no laxative prescription [black curve]). Patients receiving at least one type of laxative had a significantly higher cumulative incidence of the primary outcome than those receiving no laxatives (18% versus 7% of incidence, respectively). * *p* < 0.05 by log-rank test.

A cutoff of laxative number for predicting the primary outcome was calculated as one with a sensitivity of 0.69, a specificity of 0.54, and an area under the curve of 0.65 (the green circle represents the cutoff of laxative number in Figure 2). Patients who received at least one type of laxative (the red curve) had a significantly higher cumulative incidence of the primary outcome during the 2-year observation period after the index discharge compared with those receiving no laxatives (the black curve) (18% versus 7%, *p* < 0.001; Figure 3).

## 4. Discussion

In this analysis of our prospectively collected institutional database, we investigated the prognostic impact of constipation, which was objectively defined as the prescription of laxatives, in patients who underwent TAVR. Most of these were elderly and had multiple comorbidities, similar to the standard TAVR candidates’ cohort. Approximately half of them received at least one laxative for constipation, with a smaller percentage of patients receiving multiple laxatives. The use of laxatives correlated with higher systolic blood pressure, higher plasma B-type natriuretic peptide levels, and lower prescription rates of renin–angiotensin system inhibitors at index discharge. The number of laxative types was independently associated with an increased risk of mortality and heart failure readmission following TAVR.

### 4.1. Constipation in TAVR Candidates

Constipation is commonly encountered in elderly patients [9] and can frequently be exacerbated in the setting of medical procedures or other instances of prolonged immobility [10]. Consensus definitions of what constitutes clinical constipation as a medical diagnosis can vary [11]. According to the Rome IV criteria, functional constipation is defined as satisfaction of any two of the following features: hard stools, straining, a sensation of incomplete evacuation, use of digital maneuvers, sensation of anorectal obstruction or blockage with 25 percent of bowel movement, and reduction in stool frequency [12]. Nevertheless, most of these items are subjective, and thus a unified definition of constipation is still to be widely accepted. In the present study, we objectively defined the presence of constipation as the use of laxatives [7]. We focused on the number of drug types, instead of that of tablets, to assess the severity of constipation, because it is challenging to compare the number of tablets in each drug type.

The previous literature reported that the prevalence of constipation among healthy older adults ranges approximately between 25% and 50% [13,14]. Laxatives are used approximately by 20% of community-dwelling older adults [15]. Approximately half of the patients undergoing TAVR received laxatives in the present study. The high prevalence of laxative use may probably be due to older age, female predominance, physical inactivity, and comorbid heart failure, all of which are established risk factors for constipation [16]. It is reasonable to conclude that this comorbidity may commonly be seen in many elderly patients who also are considered for TAVR.

### 4.2. Comorbidity and Constipation

In the present study, plasma B-type natriuretic peptide levels were higher in patients who had constipation. This is not surprising, because the progression of heart failure may affect gastric motility [17]. Heart failure is associated with the stimulation of sympathetic nerve activity, reducing peristalsis. Concomitant congestion and cardiac cachexia also reduce peristalsis. Heart failure patients also often require diuretics, which may affect gastric motility. Furthermore, calcium channel blockers, which are often administered for hypertensive heart failure, also reduce peristalsis [18]. Patients with chronic heart failure sometimes restrict their fiber intake due to the risk of chronic kidney disease-related hyperkalemia [19]. Strict fiber restriction may lead to further progression of constipation.

### 4.3. Constipation and Heart Failure Readmission

Patients who received at least one type of laxative had a higher risk of heart failure readmission, even after TAVR. Interestingly, the prognostic impact of the presence of constipation was independent of the severity of baseline heart failure. Multiple types of laxatives also were associated with a higher risk of the primary outcome than the single one. Of note, one type of laxative was not significantly associated with the primary outcome. Also, given that the statistically calculated cutoff for the number of laxative types was one, the prescription of any laxatives itself should have a significant negative prognostic impact compared with no prescription.

There are multiple underlying mechanisms. Many patients who undergo TAVR have clinically significant ventricular dysfunction and pulmonary hypertension despite the correction of severe aortic valve stenosis [20]. Excessive straining due to persistent constipation may increase afterload and, in turn, worsen heart failure, particularly in individuals with such extra-valvular impairments [17,21,22]. Consistently, individuals receiving any laxatives in the present study had significantly higher systolic blood pressure at baseline, indicating incremental afterload on the left ventricle. Interestingly, the prescription rate of renin–angiotensin system inhibitors was lower in our patients with constipation. Constipation is associated with hyperkalemia due to the absorption of potassium ions from residual stool [23]. Clinicians sometimes reduce the dose of these medications to manage hyperkalemia [24]. It is difficult to ascertain causality due to the limited sample size and observational nature of this study, but intended down-titrations of heart failure medications (to manage constipation-related hyperkalemia) may also contribute to worsening clinical status and subsequent heart failure readmission.

### 4.4. Clinical Implications

Given the robust evidence of TAVR improving mortality in patients with symptomatic severe aortic stenosis over medical therapy alone, our findings do not challenge the clinical efficacy of TAVR despite the baseline comorbidity burden. Nonetheless, our findings can help inform the clinician of additional residual risk post-TAVR to assist in shared decision-making among clinicians and patients being considered for this intervention. The prognostic impact of aggressive interventions for constipation in TAVR candidates remains a future concern. The management of severe constipation might stabilize enhanced afterload on the left ventricle. An amelioration of constipation might also normalize hyperkalemia and give clinicians chances to up-titrate anti-heart failure medications such as renin–angiotensin–aldosterone system inhibitors. More detailed clinical data during follow-up should further clarify the underlying association between the presence of constipation and clinical outcomes, such as hemodynamics, echocardiography, exercise tests, quality-of-life assessments, and neuro-hormonal activity.

### 4.5. Limitations

There are limitations in this study that are worth noting. This was a single-center, retrospective study with a moderate sample size, although the prospectively collected database was robust and comprehensive. We attempted to adjust for potential confounders, but uninvestigated factors such as hemodynamics data, flail scales, and race may have affected our findings. Other fatal diseases that are associated with constipation (i.e., secondary constipation), such as Parkinson’s disease and intestinal malignancy, might also not have been diagnosed. We restricted the potential variables for including the time-to-event analysis because of relatively small event numbers and to avoid statistical multiplicity errors.

We defined the severity of constipation as the number of prescribed types of laxatives. This is objective but does not consider patients’ symptoms and signs of constipation. We did not consider the amount and type of laxatives. It is challenging to appropriately compare the doses of different types of laxatives. Nevertheless, we should state that the objective definition of constipation severity remains challenging, and further consensus is required. We did not consider the trend of laxative prescription during the observation period. We analyzed the association between constipation and clinical outcomes, and detailed causality and the underlying mechanism explaining their association remains uncertain. We did not follow several clinical parameters during the observational period, such as other clinical variables including laboratory values and repeat echocardiograms.

## 5. Conclusions

The incidence of constipation was correlated with adverse clinical outcomes post-TAVR. A therapeutic approach tailored for TAVR candidates presenting with severe baseline constipation warrants further investigation and validation through extensive, multi-center cohort studies. Future research should also explore the underlying mechanisms linking constipation to poorer outcomes in TAVR patients and assess the efficacy of various interventions aimed at mitigating these risks.

## Figures and Tables

**Table 1:** Baseline characteristics obtained at index discharge.

	Total (*N* = 344)	≥1 Laxative (*N* = 166)	No Laxative (*N* = 178)	*p*-Value
Demographics				
Age, years	85 (83, 89)	85 (83, 88)	85 (83, 89)	0.59
Men	99 (29%)	52 (31%)	47 (26%)	0.19
Body surface area, m^2^	1.38 (1.28, 1.51)	1.37 (1.28, 1.49)	1.38 (1.29, 1.52)	0.82
Systolic blood pressure, mmHg	117 (106, 128)	120 (110, 129)	115 (103, 124)	0.001 *
Pulse rate, bpm	69 (63, 78)	69 (63, 78)	70 (63, 78)	0.70
General/local anesthesia	332/12	160/6	172/6	0.57
Trans-femoral approach	344 (100%)	166 (100%)	178 (100%)	-
Comorbidity				
Hypertension	251 (73%)	115 (69%)	136 (76%)	0.086
Diabetes mellitus	61 (18%)	32 (19%)	29 (16%)	0.28
Dyslipidemia	164 (48%)	82 (49%)	82 (46%)	0.31
Atrial fibrillation				
Smoking	58 (17%)	33 (20%)	25 (14%)	0.097
History of cardiac surgery	25 (7%)	12 (7%)	13 (7%)	0.57
History of malignancy	30 (9%)	17 (10%)	13 (7%)	0.22
History of stroke	45 (13%)	17 (10%)	28 (16%)	0.085
Coronary artery disease	88 (26%)	49 (30%)	39 (22%)	0.068
Peripheral artery disease	76 (22%)	42 (25%)	34 (19%)	0.11
Laboratory data				
Hemoglobin, g/dL	10.4 (9.7, 11.1)	10.3 (9.7, 11.1)	10.4 (9.7, 11.1)	0.41
Serum albumin, g/dL	3.4 (3.0, 3.6)	3.4 (3.0, 3.6)	3.4 (3.1, 3.7)	0.34
Serum total bilirubin, mg/dL	0.5 (0.4, 0.6)	0.5 (0.4, 0.6)	0.5 (0.4, 0.6)	0.097
Serum sodium, mEq/L	139 (137, 141)	139 (137, 141)	140 (138, 141)	0.054
Serum potassium, mEq/L	4.3 (4.0, 4.6)	4.3 (4.1, 4.6)	4.3 (4.0, 4.6)	0.055
eGFR, mL/min/1.73 m^2^	48.6 (34.0, 62.1)	50.3 (37.4, 65.6)	47.4 (31.0, 59.4)	0.81
Plasma BNP, pg/mL	110 (57, 227)	119 (67, 226)	90 (54, 230)	0.042 *
Echocardiography data				
LVDd, mm	45 (41, 49)	45 (41, 50)	45 (41, 49)	0.76
LVEF, %	64 (57, 72)	65 (57, 72)	64 (57, 71)	0.72
Left atrial diameter, mm	43 (37, 48)	43 (37, 48)	42 (37, 49)	0.77
Aortic valve parameter				
Maximum velocity, m/s	2.1 (1.7, 2.4)	2.0 (1.8, 2.3)	2.1 (1.7, 2.4)	0.74
Mean pressure gradient, mmHg	10 (7, 12)	9 (7, 12)	10 (6, 12)	0.79
Valve area, cm^2^	1.38 (1.16, 1.61)	1.38 (1.16, 1.60)	1.40 (1.19, 1.63)	0.48
Medications				
Beta-blockers	138 (40%)	71 (43%)	67 (38%)	0.20
Renin–angiotensin system inhibitors	224 (65%)	100 (60%)	124 (70%)	0.043 *
Mineralocorticoid receptor antagonists	100 (29%)	45 (27%)	55 (31%)	0.26
Loop diuretics	154 (45%)	81 (49%)	73 (41%)	0.090

Baseline characteristics were obtained at index discharge after trans-catheter aortic valve replacement. eGFR, estimated glomerular filtration rate; BNP, B-type natriuretic peptide; LVDd, left ventricular end-diastolic diameter; LVEF, left ventricular ejection fraction. Continuous variables are stated as medians (25% interquartile, 75% interquartile) and compared between the two groups by the Mann–Whitney U test. Categorical variables are stated as numbers and percentages and compared between the two groups by Fischer’s exact test. * *p* < 0.05.

**Table 2:** Association between potential baseline characteristics and the primary outcome.

	Univariable Analysis		Multivariable Analysis	
	Hazard Ratio (95% CI)	*p*-Value	Hazard Ratio (95% CI)	*p*-Value
Age, years	1.04 (0.98–1.12)	0.21		
Systolic blood pressure, mmHg	0.99 (0.97–1.01)	0.36		
eGFR, mL/min/1.73 m^2^	0.99 (0.98–1.01)	0.57		
Common logarithm of plasma BNP, pg/mL	2.85 (1.38–5.91)	0.005 *	2.45 (1.11–5.41)	0.026 *
Hemoglobin, g/dL	0.85 (0.64–1.13)	0.26		
LVEF, %	1.02 (0.98–1.05)	0.36		
Atrial fibrillation	3.21 (1.54–6.70)	0.002 *	2.49 (1.17–5.31)	0.018 *
Renin–angiotensin system inhibitors	0.59 (0.30–1.14)	0.12		
Loop diuretics	2.10 (1.07–4.14)	0.031 *	1.47 (0.72–3.01)	0.30
Number of laxatives	1.80 (1.26–2.57)	0.001 *	1.83 (1.27–2.63)	0.001 *

Baseline characteristics that were potentially considered to be associated with the primary outcome were included in the Cox proportional hazard ratio regression analyses. The primary outcome was defined as death or heart failure readmission after the index discharge. Variables with *p* < 0.05 in the univariable analyses were included in the multivariable analysis. CI, confidence interval; eGFR, estimated glomerular filtration rate; BNP, B-type natriuretic peptide; LVEF, left ventricular ejection fraction. * *p* < 0.05.

## Data Availability

Data are contained within the article.

## References

[1] Kodali S.K., Williams M.R., Smith C.R., Svensson L.G., Webb J.G., Makkar R.R., Fontana G.P., Dewey T.M., Thourani V.H., Pichard A.D. (2012). Two-year outcomes after transcatheter or surgical aortic-valve replacement. N. Engl. J. Med..

[2] Leon M.B., Smith C.R., Mack M.J., Makkar R.R., Svensson L.G., Kodali S.K., Thourani V.H., Tuzcu E.M., Miller D.C., Herrmann H.C. (2016). Transcatheter or Surgical Aortic-Valve Replacement in Intermediate-Risk Patients. N. Engl. J. Med..

[3] Kolte D., Vlahakes G.J., Palacios I.F., Sakhuja R., Passeri J.J., Inglessis I., Elmariah S. (2019). Transcatheter Versus Surgical Aortic Valve Replacement in Low-Risk Patients. J. Am. Coll. Cardiol..

[4] Mack M.J., Leon M.B., Thourani V.H., Pibarot P., Hahn R.T., Genereux P., Kodali S.K., Kapadia S.R., Cohen D.J., Pocock S.J. (2023). Transcatheter Aortic-Valve Replacement in Low-Risk Patients at Five Years. N. Engl. J. Med..

[5] Auffret V., Bakhti A., Leurent G., Bedossa M., Tomasi J., Belhaj Soulami R., Verhoye J.P., Donal E., Galli E., Loirat A. (2020). Determinants and Impact of Heart Failure Readmission Following Transcatheter Aortic Valve Replacement. Circ. Cardiovasc. Interv..

[6] Sumida K., Molnar M.Z., Potukuchi P.K., Thomas F., Lu J.L., Yamagata K., Kalantar-Zadeh K., Kovesdy C.P. (2019). Constipation and risk of death and cardiovascular events. Atherosclerosis.

[7] Namiuchi S., Tanita A., Sunamura S., Onodera K., Ogata T., Noda K., Takii T., Nitta Y., Yoshida S. (2024). Effect of constipation on readmission for heart failure in patients with acute heart failure. ESC Heart Fail..

[8] Ishiyama Y., Hoshide S., Mizuno H., Kario K. (2019). Constipation-induced pressor effects as triggers for cardiovascular events. J. Clin. Hypertens.

[9] Parikh P.B., Mack M., Stone G.W., Anker S.D., Gilchrist I.C., Kalogeropoulos A.P., Packer M., Skopicki H.A., Butler J. (2024). Transcatheter aortic valve replacement in heart failure. Eur. J. Heart Fail..

[10] Higgins P.D., Johanson J.F. (2004). Epidemiology of constipation in North America: A systematic review. Am. J. Gastroenterol..

[11] Glia A., Lindberg G. (1997). Quality of life in patients with different types of functional constipation. Scand. J. Gastroenterol..

[12] Drossman D.A., Sandler R.S., McKee D.C., Lovitz A.J. (1982). Bowel patterns among subjects not seeking health care. Use of a questionnaire to identify a population with bowel dysfunction. Gastroenterology.

[13] Mearin F., Lacy B.E., Chang L., Chey W.D., Lembo A.J., Simren M., Spiller R. (2016). Bowel Disorders. Gastroenterology.

[14] Whitehead W.E., Drinkwater D., Cheskin L.J., Heller B.R., Schuster M.M. (1989). Constipation in the elderly living at home. Definition, prevalence, and relationship to lifestyle and health status. J. Am. Geriatr. Soc..

[15] Sandler R.S., Jordan M.C., Shelton B.J. (1990). Demographic and dietary determinants of constipation in the US population. Am. J. Public. Health.

[16] Ruby C.M., Fillenbaum G.G., Kuchibhatla M.N., Hanlon J.T. (2003). Laxative use in the community-dwelling elderly. Am. J. Geriatr. Pharmacother..

[17] Stewart W.F., Liberman J.N., Sandler R.S., Woods M.S., Stemhagen A., Chee E., Lipton R.B., Farup C.E. (1999). Epidemiology of constipation (EPOC) study in the United States: Relation of clinical subtypes to sociodemographic features. Am. J. Gastroenterol..

[18] Elliott W.J., Ram C.V. (2011). Calcium channel blockers. J. Clin. Hypertens.

[19] Kalantar-Zadeh K., Joshi S., Schlueter R., Cooke J., Brown-Tortorici A., Donnelly M., Schulman S., Lau W.L., Rhee C.M., Streja E. (2020). Plant-Dominant Low-Protein Diet for Conservative Management of Chronic Kidney Disease. Nutrients.

[20] Genereux P., Pibarot P., Redfors B., Mack M.J., Makkar R.R., Jaber W.A., Svensson L.G., Kapadia S., Tuzcu E.M., Thourani V.H. (2017). Staging classification of aortic stenosis based on the extent of cardiac damage. Eur. Heart J..

[21] Yang S., Yu C., Guo Y., Bian Z., Fan M., Yang L., Du H., Chen Y., Yan S., Zang Y. (2020). Bowel movement frequency and risks of major vascular and non-vascular diseases: A population-based cohort study among Chinese adults. BMJ Open.

[22] Johanson J.F., Kralstein J. (2007). Chronic constipation: A survey of the patient perspective. Aliment. Pharmacol. Ther..

[23] Hida Y., Imamura T., Kinugawa K. (2023). Constipation as a Drug-Related Adverse Effect in Patients with Hyperkalemia: Sodium Zirconium Cyclosilicate versus Conventional Potassium Binders. J. Clin. Med..

[24] Leon S.J., Whitlock R., Rigatto C., Komenda P., Bohm C., Sucha E., Bota S.E., Tuna M., Collister D., Sood M. (2022). Hyperkalemia-Related Discontinuation of Renin-Angiotensin-Aldosterone System Inhibitors and Clinical Outcomes in CKD: A Population-Based Cohort Study. Am. J. Kidney Dis..

